# Completely endoscopic reversal of vertical banded gastroplasty using magnetic compression gastro-gastric anastomosis

**DOI:** 10.1055/a-2971-4097

**Published:** 2026-10-09

**Authors:** Yung Lee, Salvador Navarrete, Hassan Siddiki, Valentin Mocanu

**Affiliations:** 1 Surgery 3710 McMaster UniversityHamiltonCanada; 2 Surgery 2569 Cleveland ClinicClevelandOhioUnited States

Vertical banded gastroplasty (VBG) was among the most common bariatric operation of the 1980s to 1990s. Outlet stenosis is a well-recognized complication, and definitive management has required conversion to Roux-en-Y gastric bypass, a reoperation carrying substantial morbidity in patients who are typically elderly, malnourished, or have hostile abdomens. Magnetic compression anastomosis has recently been used to create small-bowel anastomoses as a primary bariatric procedure 1​ 2​ 3​ ​4​ but has not been applied to reverse VBG.

Two patients underwent completely endoscopic VBG reversal by magnetic compression gastro-gastrostomy under general anesthesia ( Video 1 ). Patient 1 (71F) had progressive regurgitation and reflux, tolerating only liquids and soft solids (body mass index [BMI] 30). Patient 2 (69 women; prior ventral hernia repair, cholecystectomy, and chronic obstructive pulmonary disease) was transferred urgently with gastric outlet obstruction, malnourished (BMI 18 and albumin 3.1) and requiring nasogastric decompression and parenteral nutrition. Both had remote open VBG and were high-risk candidates for complex revisional surgery; endoscopic reversal was offered as a lower-risk alternative.

**Video 1** Completely endoscopic reversal of vertical banded gastroplasty by magnetic compression gastro-gastric anastomosis.


In Patient 1, an ultraslim gastroscope crossed the stenosis into a normal excluded stomach, and a 50-mm magnet (MagDI System, GT Metabolic Solutions) was delivered across it ( Fig. 1 ). A second ultraslim scope viewed from the pouch as the second magnet was seated; the pair coupled under direct vision with fluoroscopic confirmation (28 min; Fig. 2 ). Patient was discharged home the same day. In Patient 2, the stenosis was not traversable: retained debris was cleared and the channel dilated to 15 mm, and the distal magnet deployed in the fundus in reverse Trendelenburg (67 min, 30 clearing debris). Patient 2 underwent concurrent gastrostomy.

**Fig. 1:**
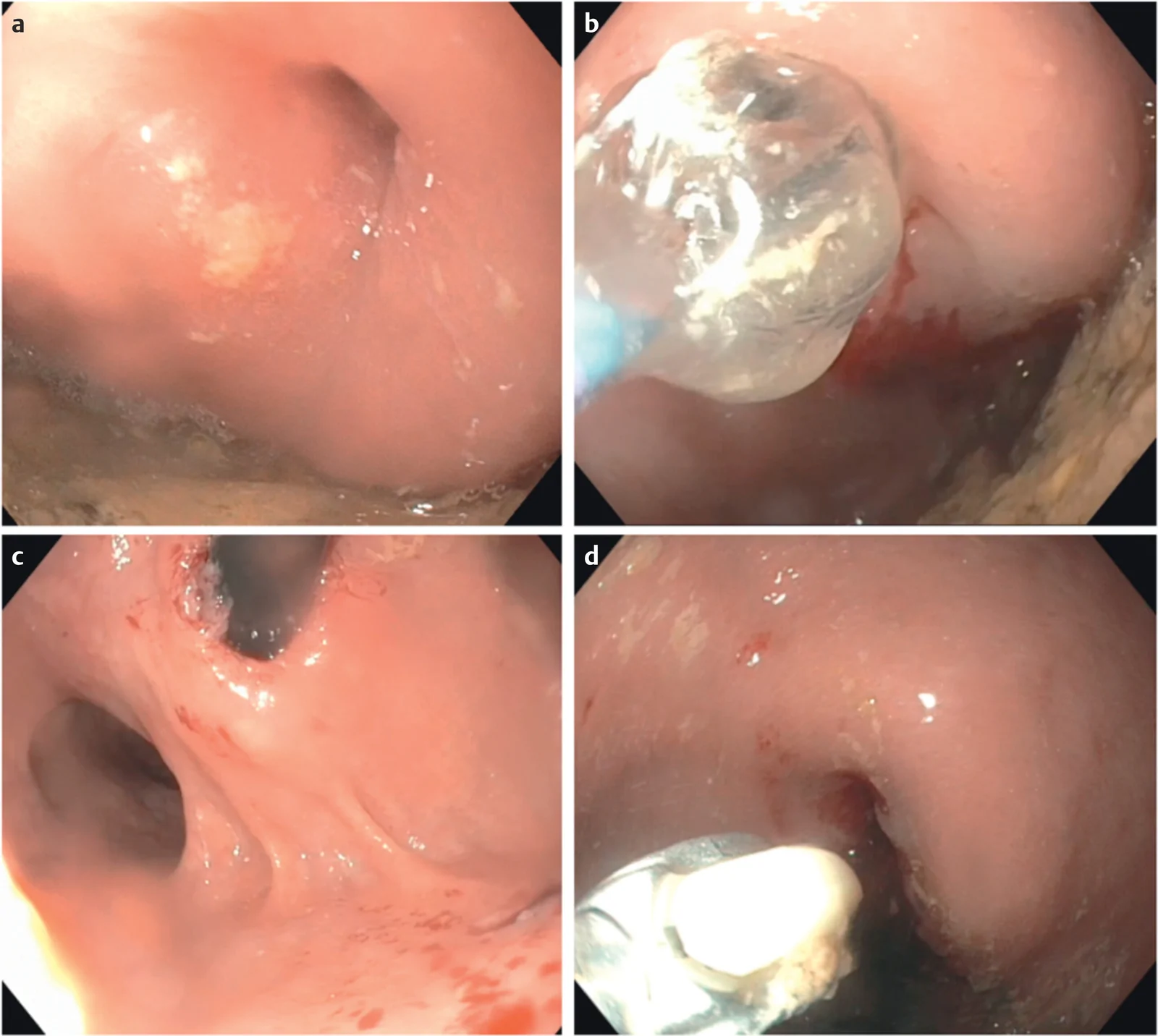
( **a** ) An endoscopic view of the vertical banded gastroplasty pouch and stenotic banded outlet. ( **b** ) Balloon dilation of the stenotic channel to 15 mm to permit the passage of the gastroscope. ( **c** ) A retroflexed view of the stenosis and the fundus. ( **d** ) Introduction of the first 50 mm magnet across the stenosis and past the gastric pouch.

**Fig. 2:**
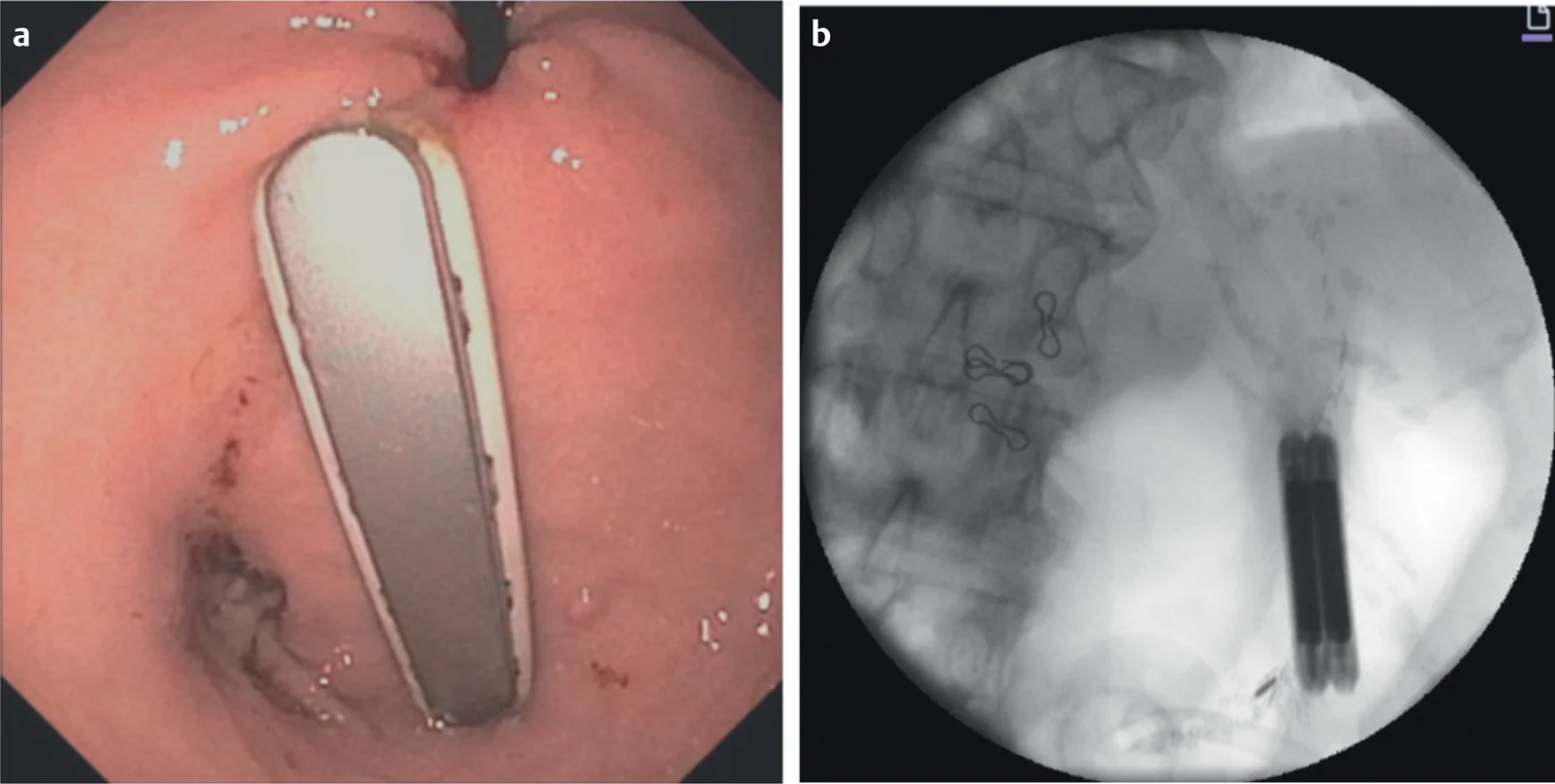
( **a** )An endoscopic view of the coupled 50-mm magnets across the gastro-gastric septum. ( **b** ) A fluoroscopic view of the coupled 50-mm magnets.

Magnets passed spontaneously, confirmed radiographically at 4 weeks, when contrast flowed freely ( Fig. 3 ) through a widely patent (~40 mm) anastomosis ( Fig. 4 ), functionally reversing the VBG. No adverse events occurred. Patient 1's symptoms resolved; Patient 2 resumed oral intake with improving nutrition.

**Fig. 3:**
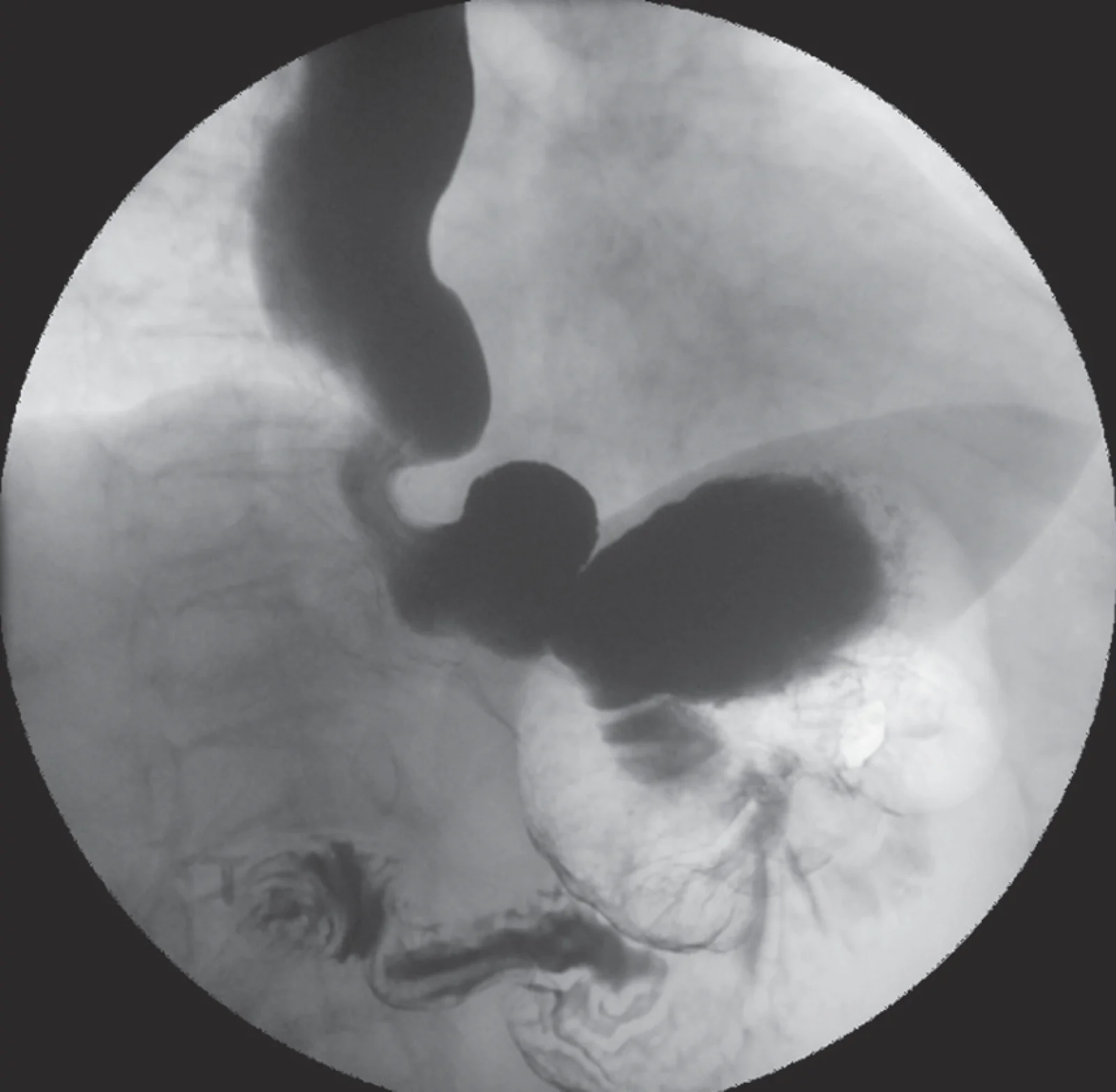
Upper gastrointestinal contrast study demonstrating flow of contrast through the newly created gastro-gastric anastomosis 4 weeks after the procedure.

**Fig. 4:**
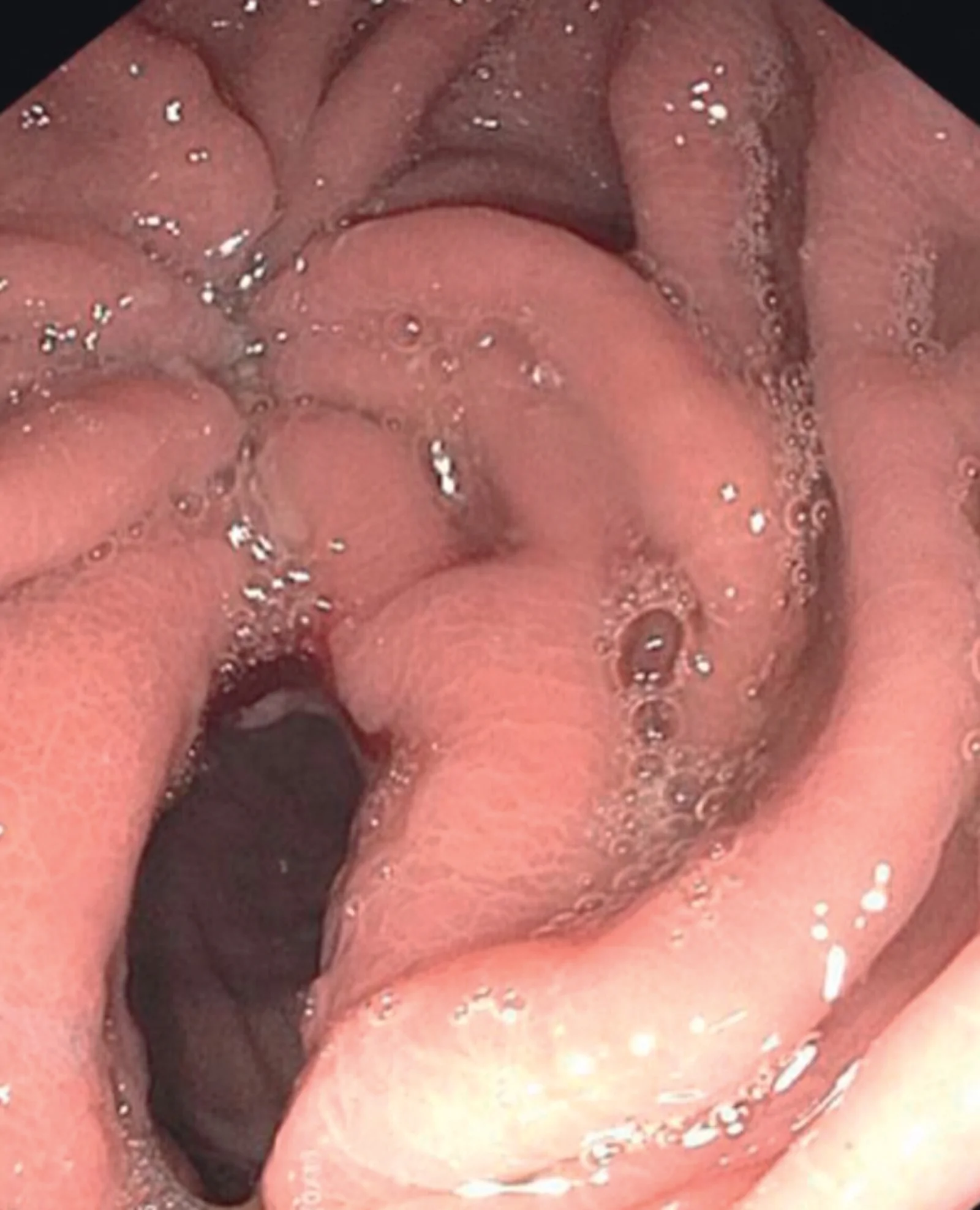
Endoscopic appearance of the matured, widely patent magnetic-compression assisted gastro-gastric anastomosis at 4 weeks.

To our knowledge, this is the first endoscopic magnetic compression gastro-gastrostomy for VBG reversal, a safe same-day endoscopic alternative to a complex surgery in frail, high-risk patients.

Endoscopy_UCTN_Code_CCL_1AB_2AC_3AD

Endoscopy_UCTN_Code_CPL_1AJ_2AF

## Declaration of GenAI Use

I confirm that all images and all components of the images were created solely by the authors of the manuscript without the use of generative artificial intelligence.
